# Ectopic Expression of *CsSUN* in Tomato Results in Elongated Fruit Shape via Regulation of Longitudinal Cell Division

**DOI:** 10.3390/ijms23179973

**Published:** 2022-09-01

**Authors:** Hao Li, Jing Han, Linjie Chen, Ni Han, Yajing Hu, Qian Ge, Zhonghai Ren, Lina Wang

**Affiliations:** State Key Laboratory of Crop Biology, Shandong Collaborative Innovation Center of Fruit & Vegetable Quality and Efficient Production, Key Laboratory of Biology and Genetic Improvement of Horticultural Crops in Huang-Huai Region, Ministry of Agriculture, College of Horticulture Science and Engineering, Shandong Agricultural University, Tai’an 271018, China

**Keywords:** *CsSUN*, heterologous expression tomato, fruit shape, cell number, cell size, seed development

## Abstract

Fruit shape, an important agronomic trait of cucumber (*Cucumis sativus* L.), is tightly controlled by a series of genes such as *CsSUN*, a homologue of *SlSUN* that is responsible for the tomato (*Solanum lycopersicum*) fruit shape via the modulation of cell division. However, the direct genetic evidence about the *CsSUN*-mediated regulation of fruit shape is still scarce, limiting our mechanistic understanding of the biological functions of *CsSUN*. Here, we introduced *CsSUN* into the round-fruited tomato inbred line ‘SN1′ (wild type, WT) via the *Agrobacterium tumefaciens*-mediated method. The high and constitutive expression of *CsSUN* was revealed by real-time PCR in all the tested tissues of the transgenic plants, especially in the fruits and ovaries. Phenotypic analyses showed that the ectopic expression of *CsSUN* increased fruit length while it decreased fruit diameter, thus leading to the enhanced fruit shape index in the transgenic tomato lines relative to the WT. Additionally, the reduction in the seed size and seed-setting rate and the stimulation of seed germination were observed in the *CsSUN*-expressed tomato. A histological survey demonstrated that the elongated fruits were mainly derived from the significant increasing of the longitudinal cell number, which compensated for the negative effects of decreased cell area in the central columellae. These observations are different from action mode of *SlSUN*, thus shedding new insights into the *SUN*-mediated regulation of fruit shape.

## 1. Introduction

Cucumber (*Cucumis sativus* L.) is a widely grown horticultural crop in the world [1,2,3,4]. Cucumber fruit size and shape, the important appearance quality traits, are determined by the fruit length (FL), fruit diameter (FD), and the ratio of fruit length to fruit diameter [5,6]. Fruit size and shape are genetically regulated by quantitative trait loci (QTLs) and, meanwhile, are subjected to the effects of environmental conditions [7,8]. To date, a series of QTLs associated with fruit size and shape have been successfully identified. For example, in a comprehensive analysis that combines three mapping populations [F_2_, F_3_ and recombinant inbred line (RIL) from Gy14 (short fruit) × 9930 (long fruit)], three developmental stages, and six growth conditions, Weng and colleagues have identified 12 consensus QTLs that are closely associated with cucumber fruit shape [7]. In a study carried out by Pan et al., two major effect QTLs (*FS1.2* and *FS2.1*) have been identified to be responsible for cucumber fruit shape, and *CsSUN* has been revealed as the candidate gene for the *FS1.2* locus [9]. It has been proposed that there are 10 candidate genes for the *FS2.1* locus, and the homolog of *SlTRM5* (TONNEAU1 Recruiting Motif) is included in particular [10]. Moreover, the functional importance of three inherited genes in the regulation of cucumber fruit shape have been uncovered by different groups. The first one is a *FRUITFULL-like* MADS-box gene, *CsFUL1*, which has been validated to control fruit elongation in Chinese long cucumbers [11]. The other two genes have been identified by the genetic analysis of two *short fruit* mutants and named as *sf1* and *sf2*, which belong to the cucurbit-specific RING-type E3 ligase family and the Histone Deacetylase Complex 1 (HDC1) family, respectively [12,13]. In a recent literature review paper by Pan et al., a total of 19 consensus QTLs and 11 QTLs were proposed to be profoundly involved in the regulation of cucumber fruit size and shape, respectively [6]. However, the molecular mechanisms underlying fruit size and shape are still largely unknown, and more QTL/genes therefore need to be identified and functionally explored.

Tomato (*Solanum lycopersicum*, 2n = 24) is an important vegetable crop worldwide [14]. Tomato displays a large divergence in fruit shape among different cultivars, varying from the small oval-shaped grape group and the blocky and squared Roma group to the large and flat beefsteak group [15]. To date, six fruit shape loci have been detected, and five candidate genes, including *sun*, *ovate*, *sov1*, *fas*, and *lc*, have been successfully cloned [9,16,17]. The *SUN* gene, which encodes a member of the plant-specific IQ67 DOMAIN (IQD) family of calmodulin-binding protein, is considered to be the major player for controlling tomato fruit shape via imposing predominant effects on fruit elongation, and thus, its overexpression can yield extremely elongated fruits [18,19,20]. The ovary and the young fruit development stages are critical for the determination of the tomato fruit shape by the *SUN* gene [21,22,23]. Evolutionary exploration demonstrates the occurrence of an unusual 24.7-kb duplication event from chromosome 10 to chromosome 7, with the aid of retrotransposon *Rider*, which locates the tomato *SUN* gene (*Solyc10g079240*) in a new genome environment and perhaps associates it with the phenotype of elongated fruits [18,19,23]. Further investigation has uncovered that the *SUN* gene stimulates tomato fruit elongation by increasing the longitudinal cell division while meanwhile inhibiting the transverse cell division [23]. Three possible molecular mechanisms have been put forward to bridge the tomato *SUN* gene and cell division. First, Clevenger et al. have reported that the *SUN* gene could influence the expression of cell division, the cell wall, and patterning-related genes at the early stage of fruit development through calcium signaling [24]. In contrast, the results from an investigation by Wang et al. demonstrate that the shifting in the transcriptional levels of the auxin signaling genes at the early stage of ovary development might play crucial roles in the regulation of cell division by the tomato *SUN* gene [25]. It has also been proposed that the tomato *SUN* gene could modulate cell division by interacting with microtubules, which are part of the cell cytoskeleton and are profoundly involved in the determination of cell division patterns [26]. In addition to elongated fruits, other abnormalities have also been observed in the overexpression lines of the tomato *SUN*, such as failure in seed development, thinner and twisted leaf rachises, and thinner stems [23].

The cucumber *SUN* (*CsaV3_1G039870*), the first identified fruit shape-related gene, has been mapped by the genetic analysis of the F_2_, F_3_ populations that are developed from WI7238 (long columnar fruit) × WI7239 (smooth and round fruit) [9]. In comparison to long-fruited WI7238, a 161-bp deletion is observed in the first exon of *CsSUN* in the round-fruited WI7239, and as a result, the expression of *CsSUN* in WI7239 is dramatically decreased [9]. However, it remains largely unknown about the genetic and molecular basis of *CsSUN*.

To unveil this ambiguity, we introduced *CsSUN* into a round-fruited tomato inbred line ‘SN1′ via the *Agrobacterium tumefaciens*-mediated method in this study. Molecular investigation demonstrated the successful expression of the *CsSUN* gene in the transgenic tomato lines. Obvious phenotypic alterations, including the elongation of fruits, the decrease in seed size and seed-setting rate, and the stimulation of seed germination, were observed in the transgenic tomato plants. Microscopic investigation further revealed the significant increase in the cell number in the longitudinal direction in the central columellae of the transgenic fruits, indicating that fruit shape control by *CsSUN* might be mainly dependent on the modulation of longitudinal cell division. These observations are different from the tomato *SUN* gene that controls fruit shape via affecting cell division in both the transverse and the longitudinal directions. Altogether, this study expands our knowledge about the genetic and molecular basis of the *SUN/IQD* gene and could provide a potential target for future molecular breeding.

## 2. Results

### 2.1. Sequence Feature Comparison between CsSUN and SlSUN

**Figure 1:**
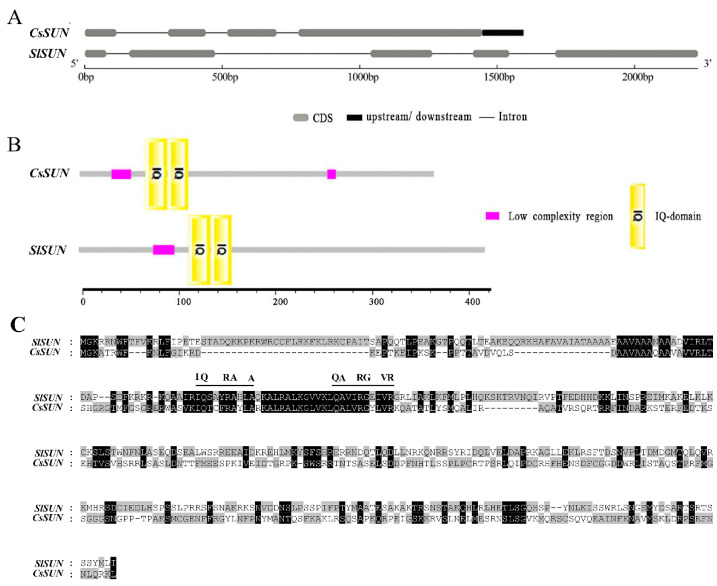
Gene and protein structures of *CsSUN* and *SlSUN*. (**A**) Gene structure comparison between *CsSUN* and *SlSUN* genes. (**B**) Conservative domains in SlSUN and CsSUN proteins. (**C**) Amino acid sequence comparison between SlSUN and CsSUN. The IQ domains are labelled on the top of corresponding amino acids with black lines.

*CsSUN*, a homologue of tomato *SUN* (*SlSUN*), was the first reported gene to regulate cucumber fruit shape. Gene structure analysis showed that *CsSUN* was 1446 bp in length and contained four exons and three introns, while *SlSUN* was 2230 bp in length and included five exons and four introns (Figure 1A). CsSUN, a member of the IQ67-domain (IQD) protein family, was predicted to contain two conserved IQ domains and two low complexity regions by the SMART online tool (Figure 1B,C). This protein displayed 65.269% similarity with SlSUN, and the core motifs of its IQ domains were “IQTCFRAYLA” and “QALVRGYLVR”, respectively (Figure 1C). In contrast, SlSUN contained two IQ domains and one low complexity region (Figure 1B). The core motifs of the two IQ domains in the SlSUN protein were “IQSAYRAHLA” and “QAVIRGEIVR”, respectively (Figure 1C). Previous studies showed that the tomato *SUN* has widespread effects not only on fruit shape but also on leaf and stem morphology, and a positive relationship was revealed between the *SUN* expression level and tomato fruit elongation as well [21,22]. The observed divergence in sequence features between *CsSUN* and *SlSUN* prompted us to further explore the functions of *CsSUN* in the regulation of organ morphology and particularly fruit shape.

### 2.2. Identification of CsSUN Transgenic Lines

**Figure 2:**
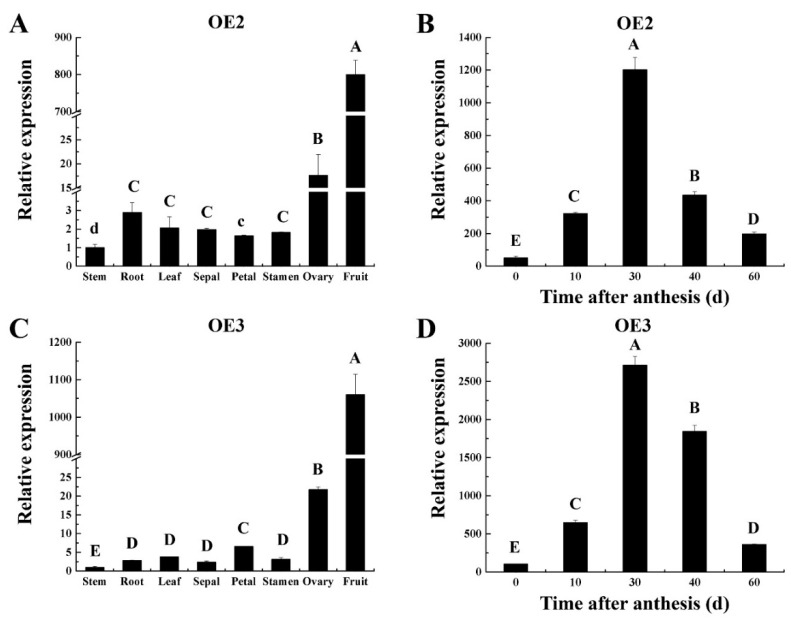
Temporospatial analysis of *CsSUN* expression in transgenic tomato lines by qRT-PCR. (**A**,**C**) *CsSUN* expression in different tissues from two transgenic tomato lines OE2 (**A**) and OE3 (**C**). (**B**,**D**) *CsSUN* expression in the fruits from OE2 (**B**) and OE3 (**D**) at different developmental stages. Data are displayed as mean ± standard deviation of three biological repeats. Different capital and lower letters indicate significant differences at the statistical levels of 0.01 and 0.05, respectively (unpaired two-tailed *t*-test).

To unveil the biological functions of the *CsSUN* gene, we carried out the genetic transformation for tomato plants. pROK II-*CsSUN*, wherein the *CsSUN* gene was driven by a CaMV35S promoter, was introduced into the tomato inbred line ‘SN1’ via the *Agrobacterium*-mediated method. After shoot and root induction, the yielded seedlings were transferred to the soil. Transgenic validation was carried out by PCR with genomic DNAs from the regenerated tomato seedlings, and three OE-*CsSUN* transgenic lines (OE1, OE2 and OE3) were obtained. We selected two transgenic lines, OE2 and OE3, and grew them to a T_3_ generation via self-crossing for subsequent analysis. The expression of *CsSUN* was first detected by qRT-PCR with the T_3_ plants of OE2 and OE3. The results showed that *CsSUN* was ectopically expressed in the roots, stems, leaves, sepals, petals, stamens, ovaries, and fruits of both transgenic tomato lines in comparison to the WT, wherein no detectable expression signals were identified (Figure 2A,C). The highest expression level of the *CsSUN* gene was observed in the fruits, followed by the ovaries and the main organs wherein the endogenous *SUN* gene functions (Figure 2A,C). We further analyzed the expression pattern of the *CsSUN* gene in the transgenic fruits at different developmental stages. *CsSUN* transcription fluctuated over the investigation period and the highest level was observed at 30 DAA for both transgenic lines (Figure 2B,D).

### 2.3. Ectopic Expression of CsSUN Results in Elongated Tomato Fruits

**Figure 3:**
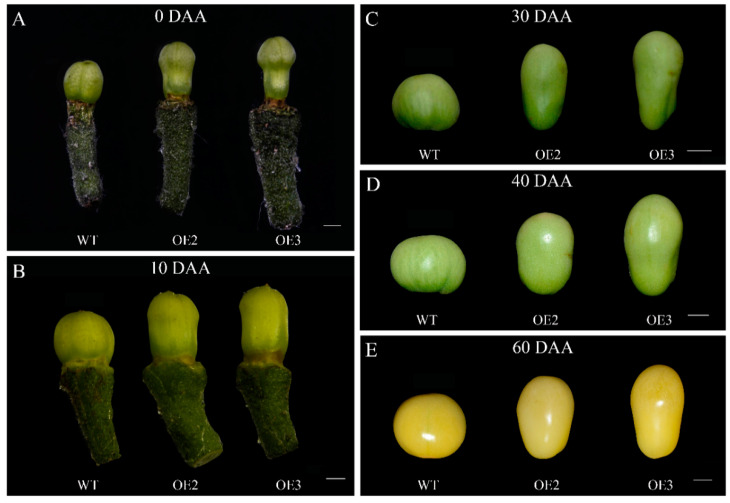
Morphological variations in tomato fruits between wild type plants and transgenic lines. (**A**) Tomato ovaries from wild type (WT) plants and two transgenic lines OE2 and OE3 at 0 days after anthesis (0 DAA). (**B**–**E**) Tomato fruits from WT, OE2, and OE3 at 10 DAA (**B**), 30 DAA (**C**), 40 (**D**), and 60 DAA (**E**). Scale bars represent 1 mm in (**A**,**B**) and 1 cm in (**C**–**E**), respectively.

**Figure 4:**
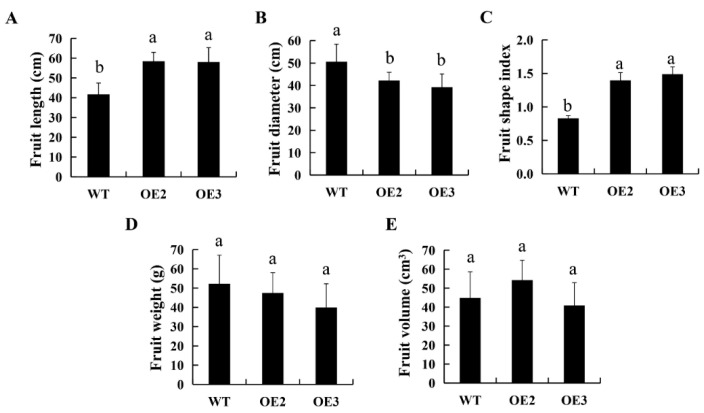
Variations in phenotypic parameters of tomato fruits between wild type plants and transgenic lines. (**A**–**E**) Comparison of fruit length (**A**), fruit diameter (**B**), fruit shape (**C**), fruit weight (**D**), and fruit volume (**E**) between wild type (WT) plants and two transgenic lines OE2 and OE3. Fruits from WT, OE2, and OE3 at 60 days after anthesis were used for determination of phenotypic parameters. Data are displayed as mean ± standard deviation of 50 fruit samples. Different lower letters indicate significant differences at the statistical level of 0.05 (unpaired two-tailed *t*-test).

We introduced cucumber *SUN* into tomato ‘SN1’ plants to explore whether the fruit shape could be influenced by the ectopic expression of the *CsSUN* gene. As shown in Figure 3, the elongated ovaries/fruits were observed at developmental stages for the *CsSUN*-overexpressed lines. In comparison to the WT, the fruit lengths of OE2 and OE3 were increased by 40% and 39.3%, respectively, while the fruit diameters of OE2 and OE3 were decreased by 16.6% and 22.5%, respectively, at 60 DAA (Figure 4A,B). As a result, the significantly increased fruit shape index was observed for both transgenic tomato lines (Figure 4C). In contrast, no significant differences in fruit weight and fruit volume were revealed between the transgenic lines and the WT (Figure 4D,E), indicating that alterations in fruit shape might be attributed to mass rearrangement (Figure 4).

**Figure 5:**
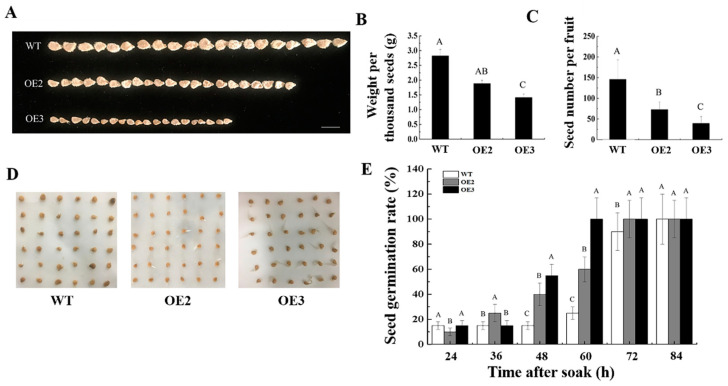
Variations in seed size and germination rate between wild type tomato and transgenic lines. (**A**) Comparison of seeds size between wild type (WT) plants and two transgenic lines OE2 and OE3. (**B**,**C**) Statistical analysis for variations in two seed-related parameters, weight per thousand seeds (**B**) and seed number per fruit (**C**), between WT, OE2, and OE3. (**D**) Comparison of seed germination rate between WT, OE2, and OE3. (**E**) Statistical analysis for variations in seed germination rate between WT, OE2, and OE3. Data are displayed as mean ± standard deviation of five biological repeats in (**B**), of 20 fruit samples in (**C**), and of six independent experimental repeats in (**E**). Different capital letters indicate significant differences at the statistical level of 0.01 (unpaired two-tailed *t*-test).

Intriguingly, we found that the seed size, weight, and seed-setting rate were significantly decreased, while the initiation of the seed germination was stimulated for the transgenic tomato lines in comparison to the WT plants (Figure 5).

### 2.4. Ectopic Expression of CsSUN Alters Both Cell Division and Size in Transgenic Tomato Fruits

**Figure 6:**
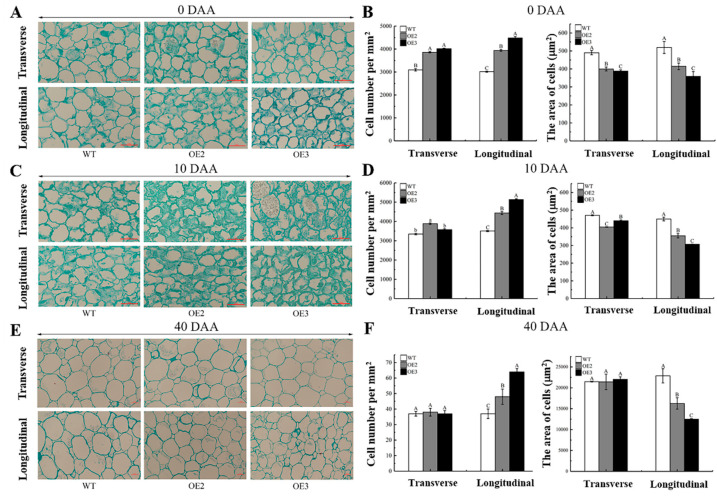
Cell morphology in the central columellae of tomato fruits from wild type plants and transgenic lines. (**A**,**C**,**E**) Microscopic view of fruit central columella cells in transverse (top panel) and longitudinal directions (lower panel) from wild type (WT) plants and two transgenic lines OE2 and OE3 at 0 days after anthesis (0 DAA, (**A**)), 10 DAA (**C**) and 40 DAA (**E**). Scale bars represent 25 μm in (**A**,**C**) and 100 μm in (**E**), respectively. Three biological repeats were performed. (**B**,**D**,**F**) Cell number (left panel) and area (right panel) in transverse and longitudinal directions in the central columellae of tomato fruits from WT, OE2, and OE3 at 0 DAA (**B**), 10 DAA (**D**), and 40 DAA (**F**), respectively. In (**B**,**D**,**F**), data are displayed as mean ± standard deviation of three biological repeats in the left panels and of at least 120 cells in the right panels. Different capital and lower letters indicate significant differences at the statistical levels of 0.01 and 0.05, respectively (unpaired two-tailed *t*-test).

Plant tissues and organs are composed of cells, and their size is closely related to cell number and structure [5]. To unveil whether the elongated fruits were derived from alterations in cell number or/and morphology, we investigated the number and size of the fruit central columella cells from the transgenic tomato lines at different developmental stages. In the transverse direction, there were no significant differences in cell number and area of the fruit central columellae between the transgenic lines and the WT plants at 40 DAA, though the fluctuations in the two parameters were observed at 0 DAA and 10 DAA (Figure 6A–F). In the longitudinal direction, we observed that the cell number was constantly higher, and the cell area was constantly lower in the fruit central columellae from the transgenic lines than those in the WT plants over the investigation period (Figure 6). It was noted that the diameter of the transgenic fruits was significantly decreased, while in the transverse direction, the cell number and area in the fruit central columellae of the transgenic lines was similar to the WT, implying that the transverse cell division might be decreased to some extent in the central columellae of transgenic fruits (Figure 4B and Figure 6).

## 3. Discussion

In this study, we introduced a fruit shape-related major-effect QTL *CsSUN* into tomato ‘SN1’ plants under the constitutive 35S promoter, and the elongated ovaries/fruits were observed at developmental stages for the *CsSUN*-overexpressed lines (Figure 3). Previous studies have documented the positive relationship between *SlSUN* expression and fruit elongation in tomato plants [18,20,23,27]. For example, the elongated fruits have been reported in the tomato near-isogenic lines (NILs) that carry the *SlSUN* duplication relative to the WT plants with round fruits (LA1589ee and LA1589pp; Sun1642ee and Sun1642pp), possibly due to the enhanced expression of *SlSUN* in the vascular tissues of sepals and ovaries during embryo development, and in the sepals, petals, and ovaries at the anthesis stage in the NILs [23]. Moreover, the 35S-driven overexpressor of *SlSUN* in the round-fruited background (Sun1642ox and LA1589ox) leads to the extreme increasing of tomato fruit length, together with abnormal proximal end elongation and pronounced tips, as well as failure in seed development [23]. Wu et al. have also reported that *SlSUN*-mediated regulation of fruit shape involves both the stimulation of longitudinal cell division and the repression of transverse cell division in *sun* NILs [23]. It should be noted that although both *CsSUN* and *SlSUN* are involved in the regulation of fruit shape, the closest homologue relationship has been revealed with *SlSUN32* for *CsSUN* and with *CsSUN13* for *SlSUN* [28]. Consistently, our observations suggest that *CsSUN* functions primarily in longitudinal cell division during fruit shape regulation. The divergence in sequence features and phylogenetics between *CsSUN* and *SlSUN* may be one of the main reasons for their different functions in the regulation of organ morphology and particular fruit shape, reflecting differences in gene function in species evolution. Furthermore, there remains no evidence about the biological functions in the regulation of fruit shape for *CsSUN13* and *SlSUN32.* Both of them therefore deserve further investigation in the future.

**Figure 7:**
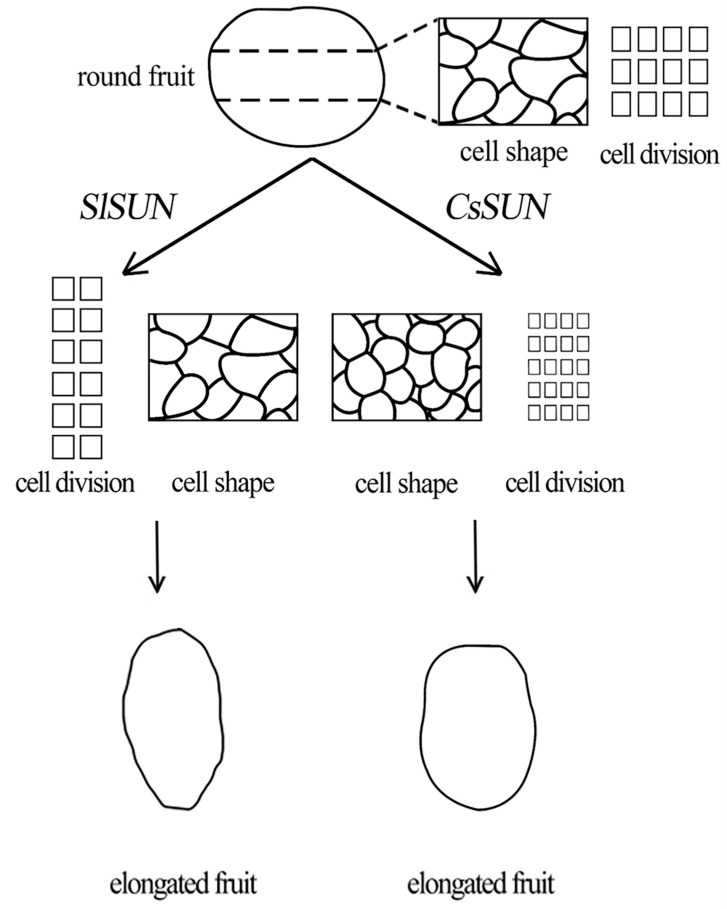
A proposed model to explain the *CsSUN*-mediated regulation of fruit shape. Both *SlSUN* and *CsSUN* are involved in controlling fruit shape, while the underlying mechanisms are different. For the *SlSUN* gene, its genome duplication in tomato leads to the elongated fruits via strikingly stimulating cell division in longitudinal direction and inhibiting cell division in transverse direction without significant influences on cell size [23]. In contrast, the elongated fruits by the ectopic expression of *CsSUN* gene in tomato mainly derive from the stimulated cell division in longitudinal direction with relatively weak influences on transverse cell division, though the cell expansion and size were significantly decreased in longitudinal direction in the central columellae.

Integrating our observations in this study with the previously described conclusions [23], we proposed a model to explain the difference in fruit shape regulation between the *SlSUN* and *CsSUN* genes (Figure 7). For the *SlSUN* gene, its genome duplication in tomato imposes opposite effects on cell division: strong stimulation of the longitudinal cell division and inhibition of the transverse cell division without significant influences on cell size; as a result, the fruits of the genome duplicated-*SlSUN* tomato plant are elongated. In contrast, the ectopic expression of the *CsSUN* gene in the tomato leads to the elongated fruits, mainly due to the enhanced cell division in the longitudinal direction with relatively weak inhibitory effects on transverse cell division, though the decreased cell size was also observed.

In addition to the significant increase in fruit length, the elongated cotyledons, and the leaves, the thinned and twisted stems were observed for the *SlSUN* overexpression plants under the 35S promoter, suggesting its pleiotropic influences on plant morphology [23]. In the present study, although the *CsSUN* gene was expressed in the roots, stems, leaves, sepals, petals, and stamens of the OE2 and OE3 plants as well (Figure 2A, C), no significant phenotypic alterations were observed. Notably, the seed size, weight, and seed-setting rate were significantly decreased, while the initiation of seed germination was stimulated for the 35S-driven transgenic tomato lines in comparison to the WT plants (Figure 5). These observations were distinct from the *SlSUN* gene, again reflecting their functional divergence. The development and germination of the seed are regulated by multiple factors. For example, ABA is a key hormone in the development and maturation of the seed [29]. ABA and ethylene act antagonistically in the process of seed germination, with ABA suppressing germination and ethylene promoting it [30,31,32]. Our results suggest that *CsSUN* plays important roles in the development and germination of the seed, but the molecular mechanism regulating seed development and germination by *CsSUN* needs further studies.

In summary, the results in the present study demonstrated that the *CsSUN* gene might function as a pleiotropic effector in the growth and development of tomato plants. Future studies, such as the genetic transformation and functional analysis of *CsSUN* in cucumber, are needed to better understand the biological significance of *CsSUN*.

## 4. Materials and Methods

### 4.1. Plant Materials and Growth Conditions

The germinated seedlings of cucumber (*Cucumis sativus* L.cv. ‘CNS21’), wild type (WT) tomato (*Solanum lycopersicum* cv. ‘SN1’) and transgenic tomato were first grown in a growth chamber under a 16 h light period with an air temperature of 25 °C and 8 h darkness with an air temperature of 18 °C. When they were grown to two-leaf or three-leaf stages, the cucumber and tomato seedlings were transferred to the greenhouse of Shandong Agricultural University, Tai’an, Shandong Province, P.R. China. Regular field managements were carried out over the cultivation period. At about 70 days after sowing, the main roots and stems, mature leaves, sepals, petals, stamens, and ovaries were collected from the WT and transgenic tomato plants. Fruits were sampled at 0 days after anthesis (DAA), 10 DAA, 30 DAA, 40 DAA, and 60 DAA, respectively, from the WT and transgenic tomato plants. ‘CNS21’, the Northern-China type cucumber inbred line, has long been the commercial fruit with the average fruit shape index (the ratio of length to diameter) over 10.0. The tomato inbred line ‘SN1’ sets round commercial fruit with the average fruit shape index below 1.0.

### 4.2. Sequence Analysis and Cloning of CsSUN Gene

The sequences of *CsSUN* (*CsaV3_1G039870*) and *SlSUN* (*Solyc10g079240*) were downloaded from the Cucurbit Genomics Database (http://cucurbitgenomics.org/organism/20, accessed on 21 December 2020) and the Solanaceae Genomics Network (https://solgenomics.net/, accessed on 21 December 2020), respectively. Gene structure analysis was performed using the Gene Structure Display Server (GSDS) (http://gsds.cbi.pku.edu.cn/, accessed on 22 December 2020). Conserved motifs of *CsSUN* and *SlSUN* were obtained by using the SMART online tool (http://smart.embl-heidelberg.de/smart/set_mode.cgi?NORMAL = 1#, accessed on 23 December 2020). Alignment and similarity analysis for amino acid sequences of CsSUN and SlSUN were performed with Clustal X 1.83 and GeneDoc software.

Young leaves from the cucumber inbred line ‘CNS21’ were used for total RNA extraction according to the method of Zhang et al. [33]. The quality of the RNA samples was first checked by agarose gel electrophoresis, and then, 1 µg of total RNAs was aliquoted for first-strand cDNA synthesis using TransScript^®^ II One-Step gDNA Removal and cDNA Synthesis SuperMix (TransGen Biotech, Beijing, China), according to the manufacturer’s instructions. The *CsSUN* gene was amplified by TransStart^®^ KD Plus DNA Polymerase (TransGen Biotech, Beijing, China) using an ABI PCR machine (Thermo Fisher Scientific, Waltham, MA, USA). The primers used for *CsSUN* cloning are listed in Table 1.

### 4.3. Binary Vector Preparation and Tomato Plant Transformation

The coding region of *CsSUN* was introduced into the pEASY vector using pEASY-Blunt Simple Cloning Kit (TransGen Biotech, Beijing, China) for sequencing validation. After digestion of the validated plasmid DNAs by XbaI/KpnI, the target fragments were recovered and further cloned into binary vector pROKII under the cauliflower mosaic virus (CaMV) 35S promoter. The pROK II-*CsSUN* was introduced into tomato ‘SN1’ plants by the *Agrobacterium tumefaciens*-mediated method, as previously described [34]. The transgenic events were revealed by PCR with genomic DNAs, and the ectopic expression of the cucumber *SUN* gene was further verified by quantitative real-time PCR (qRT-PCR) with total RNAs from the transgenic tomato plants. T_3_ transgenic plants were used for subsequent research.

### 4.4. Expression Analysis of CsSUN by qRT-PCR

Total RNAs were extracted from different tissues [main roots and stems, sepals, petals, stamens, ovaries (fruits at 0 DAA), as well as fruits at 60 DAA] of T_3_ transgenic plants and the cDNAs were synthesized from the total RNAs according to the method described in Section 2.2. qRT-PCR for the *CsSUN* gene was performed using the 2 × M5 HiPer SYBR Premix EsTaq (Mei5 Biotechnology, Beijing, China) on the ABI 7500 Real-Time PCR System (Applied Biosystems, Waltham, MA, USA), according to the manufacturer’s instructions, with the tomato *β*-*actin* gene used as an internal control. The PCR procedure was 95 °C for 30s, 40 cycles of 95 °C for 30 s, 55 °C for 15 s, and 72 °C for 15 s. Three biological repeats were performed for each sample. The relative gene expression was calculated with the 2^−∆∆Ct^ method [35]. The primers used for qRT-PCR are provided in Table 1.

### 4.5. Phenotypic Characterization of Transgenic Tomato Fruits

Fruits were sampled from a WT and two transgenic lines (OE2 and OE3) at 0 DAA, 10 DAA, 30 DAA, 40 DAA, and 60 AA for photographing. Using samples at 60 DAA, several crucial parameters were further determined for evaluation of the phenotypic variations: (1) fruit length and diameter were measured with Vernier calipers, and the fruit shape index (the ratio of length to diameter) was then calculated accordingly; (2) individual fruit weight was determined by an electronic balance; (3) a metering tank was used to determine the volume of individual fruit by calculating the alteration in water volume before and after immersing the fruit. In total, 50 biological repeats were carried out for each parameter.

### 4.6. Microscopic Investigation of Transgenic Tomato Fruits

Cell morphology in the fruit central columellae from the WT, OE2, and OE3 plants was analyzed using a NIKON Eclipse Ni upright microscope (Nikon Corporation, Tokyo, Japan) at 0, 10, and 40 DAA, respectively, according to the method of Zhang et al., with some modifications [13]. After being fixed, the middle parts of the central columellae were dissected from the fruit samples and embedded in paraffin. Then 4 μm-thick sections were generated in the transverse and longitudinal directions for staining and photographing. Cell size and number per unit area were determined using Image J software (v. 1.8.0, NIH, New York, NY, USA).

### 4.7. Data Analysis

The data were analyzed with statistical algorisms installed in Microsoft Excel 2016 and presented as mean ± standard deviation. Significant difference was evaluated for all comparisons at the statistical level of 0.01 or 0.05.

## 5. Conclusions

Here, we introduced *CsSUN* into the round-fruited tomato inbred line ‘SN1’ via the *Agrobacterium tumefaciens*-mediated method. The results of the real-time PCR showed that *CsSUN* were ectopically expressed in all the tested tissues of the transgenic plants, especially in the fruits and ovaries. The highest transcription level of *CsSUN* was observed in the fruits at 30 DAA for both transgenic lines. The increase in fruit length and the decrease in fruit diameter were clearly observed in the transgenic tomato plants. The histological analysis demonstrated that the significant increase in cell number in the longitudinal direction in the central columellae was the main cause of the elongated fruits. Meanwhile, the decrease in seed size and the seed-setting rate and the stimulation of the seed germination were observed in the transgenic tomato plants relative to the WT. These results are different from the action mode of *SlSUN*. Our study not only expanded and deepened the insight into the *SUN*-mediated regulation of fruit shape, it also provided important clues for further studies.

## Figures and Tables

**Table 1:** Primers used in this study.

Primer Name	Forward Primer	Reverse Primer
CsSUN-cloning	GGATCCATGGGGAAAGCAACGAGATG	ACTAGTTCATAATTTTCTCTGCAAAT
β-Actin	TGTTGCTATTCAGGCTGTGC	CTGCTCCTGGCAGTTTGAAT
CsSUN-RT	GCACTTAGAGCTTTGAAAGG	CTACTGTGAACGGAAACTGTG

## Data Availability

Not applicable.
